# Use of Abortion Services in Massachusetts After the *Dobbs* Decision Among In-State vs Out-of-State Residents

**DOI:** 10.1001/jamanetworkopen.2023.32400

**Published:** 2023-09-06

**Authors:** Brianna Keefe-Oates, Isabel Fulcher, Jennifer Fortin, Alisa B. Goldberg, Jarvis T. Chen, Barbara Gottlieb, Elizabeth Janiak

**Affiliations:** 1 Department of Social and Behavioral Sciences, Harvard TH Chan School of Public Health, Boston, Massachusetts; 2 Department of Research, Planned Parenthood League of Massachusetts, Boston; 3 Department of Medicine, Harvard Medical School, Boston, Massachusetts

## Abstract

This cohort study compares observed vs expected abortion counts after Dobbs in Massachusetts among in-state vs out-of-state residents.

## Introduction

The *Dobbs v Jackson Women’s Health Organization* decision in June 2022 overturned the federal right to provide abortion care, precipitating rapid changes in state laws. Initial reports after *Dobbs* found that interstate travel for abortion increased, likely resulting in increased costs and logistical burdens for many individuals. Increases in interstate travelers have primarily occurred in permissive states neighboring areas with increased restrictions or new bans.^1^ Given the large and increasing number of people traveling for abortion, states not neighboring low- or no-access areas may also experience increases in out-of-state patients. This study aimed to assess pre- vs post-*Dobbs* trends in abortion care use and charitable funding among in- vs out-of-state residents receiving abortion care in Massachusetts, a state with few abortion restrictions but distant from states with total bans.

## Methods

In this cohort study, we conducted a retrospective analysis of electronic medical records of people receiving abortion care January 2018 through October 2022 at Planned Parenthood League of Massachusetts (PPLM), which provides more than 50% of abortions in Massachusetts.^2^ We used previously published time-series methods to estimate expected numbers of abortions before and after *Dobbs* while accounting for annual trends, seasonality, and the COVID-19 pandemic.^3^ We compared observed abortion counts in the post-*Dobbs* period to expected counts had the *Dobbs* decision not occurred, examining total abortions and abortions provided to out-of-state residents. Using January 2018 to June 2022 as the baseline period, we used baseline patterns to calculate expected counts after *Dobbs*. We calculated absolute and percentage deviations between observed and expected counts using negative binomial models and bootstrapping to calculate prediction intervals (PIs).

Separately, a linear probability model estimated changes from before to after *Dobbs* in the percentage of out-of-state vs in-state residents receiving abortion funding, using July to October yearly data. We included covariates to control for seasonality and an interaction term between in- vs out-of-state status and pre- vs post-*Dobbs* period. This study follows the STROBE reporting guideline and was approved by the Mass General Brigham Institutional Review Board.

## Results

The sample included 45 797 abortions, including 44 153 among in-state and 1538 among out-of-state residents. In the first 4 months after *Dobbs*, most travelers came from other New England states. Travelers were also from non–New England states, including states with increasing abortion restrictions, such as Florida and Texas. After *Dobbs*, there was a 6.2% (95% PI, 0.3%-12.9%) increase in the total number of abortions above the expected, equivalent to an estimated 190 additional abortions overall. During that period, there was an estimated 37.5% (95% PI, 7.8%-79.4%) increase in the number of abortions among out-of-state residents above expected, an estimated 45 additional abortions among out-of-state residents (Table 1). While the estimated percentage of in-state residents receiving abortion funding increased from 1.9% to 3.1%, the estimated percentage of out-of-state travelers receiving funding increased significantly more, from 8.4% to 18.3% (*P* = .01) (Table 2).

**Table 1.** Monthly Deviations in Abortions, July-October 2022^a^

Month	Abortions provided, No.	Difference between observed and expected, No. (95% PI)	Percentage change from expected (95% PI)
**Among all patients**			
July	865	96 (16 to 170)	12.5 (1.9 to 24.5)
August	811	21 (−63 to 96)	2.7 (−7.2 to 13.4)
September	811	81 (5 to 157)	11.1 (0.06 to 24.0)
October	750	4 (−1 to −84)	−0.01 (−10.1 to 10.8)
Total	3237	190 (9 to 369)	6.2 (0.3 to 12.9)
**Among out-of-state residents**			
July	47	17 (2 to 28)	56.7 (4.4 to 147.4)
August	23	−9 (−25 to 3)	−28.1 (−52.1 to 15.0)
September	45	16 (2 to 28)	55.2 (4.7 to 164.7)
October	40	22 (8 to 33)	78.6 (19.1 to 194.1)
Total	155	45 (12 to 73)	37.5 (7.8 to 79.4)

^a^ Negative binomial models adjusted for the number of days abortions were offered at Planned Parenthood League of Massachusetts each month, the acute COVID-19 period (March 2020 through April 2021), monthly linear time trends, and harmonic terms to control for seasonality.

**Table 2.** Estimated Proportion of Patients Using Abortion Funding, July to October 2018-2022

Patient residence	Patients (N = 15 275)			
	Unadjusted, No. (%)		Adjusted, % (95% CI)^a^	
	Before *Dobbs*	After *Dobbs*	Before *Dobbs*	After *Dobbs*
In state	231 (2.5)	121 (3.8)	1.9 (1.18-2.7)	3.1 (2.4-3.9)
Out of state	29 (9.5)	30 (19.0)	8.4 (6.6-10.2)	18.3 (15.7-20.9)

^a^ Model adjusted for acute COVID-19 period and month and year fixed effects. Standard robust errors were used to calculate CIs.

## Discussion

This cohort study found that after *Dobbs,* the number of patients traveling to Massachusetts for abortion care at PPLM increased despite Massachusetts not bordering any state with an abortion ban. We also observed increased use of charitable funding for abortion among out-of-state residents. Although we cannot rule out that these results were not associated with *Dobbs*, the rapid and disproportionate increase in out-of-state patients at the largest provider of abortion care in Massachusetts may reflect initial changes associated with the *Dobbs* decision in states distant from restrictive states.

Limitations of this study include the small post-*Dobbs* sample size leading to imprecise estimates with wide PIs. Although it is possible that the increase was limited to PPLM, preliminary counts statewide showed an increase in abortion after *Dobbs*; thus we do not suspect that PPLM’s experience was unique.^1^

Given the numerous financial, logistical, and emotional burdens that travel for abortion causes, it is crucial to understand changes in nonban states. As new data become available, methods like ours may promptly identify areas with changing patient needs. This may help the abortion service infrastructure adapt to the new legal landscape and provide supportive, prompt access for all patients seeking abortion regardless of their state of residence.^4,5,6^

## Notes

Supplement.
Data Sharing Statement
