# Supplementary material for: Use of Abortion Services in Massachusetts After the Dobbs Decision Among In-State vs Out-of-State Residents
Source: JAMA Netw Open. 2023 Sep 6;6(9):e2332400. doi: 10.1001/jamanetworkopen.2023.32400 (PMC10483311; doi:10.1001/jamanetworkopen.2023.32400)
Supplement: Supplement. — Data Sharing Statement [file jamanetwopen-e2332400-s001.pdf]

Data Sharing Statement

Keefe-Oates. Use of Abortion Services in Massachusetts After Dobbs Decision Among In-State vs Out-of-State Residents. JAMA Netw Open. Published online September 6, 2023. doi:10.1001/jamanetworkopen.2023.32400

Data

Data available: No

Additional Information

Explanation for why data not available: Data will not be made available.
